# Therapeutic Targeting of Hepatic Stellate Cell Inactivation and Collagen Degradation by IC-2 Mitigates Liver Fibrosis

**DOI:** 10.3390/ijms27219522

**Published:** 2026-10-24

**Authors:** Noriko Itaba, Ken-ichiro Abe, Yohei Kono, Hiroki Shimizu, Hiroyuki Oka, Minoru Morimoto, Daisuke Nanba, Goshi Shiota

**Affiliations:** 1 Division of Regenerative Medicine and Therapeutics, Department of Genomic Medicine and Regenerative Therapy, School of Medicine, Faculty of Medicine, Tottori University, Yonago 683-8503, Tottori, Japan; k-abe@okayama-u.ac.jp (K.-i.A.); dnanba@tottori-u.ac.jp (D.N.); gshiota@tottori-u.ac.jp (G.S.); 2 KanonCure Inc., Yonago 683-8503, Tottori, Japan; 3 Research Initiative Center, Tottori University, Tottori 680-8550, Tottori, Japanmorimoto.m.6e04@m.isct.ac.jp (M.M.)

**Keywords:** liver fibrosis, Wnt/β-catenin signal inhibitor, hepatic stellate cells activation, therapeutic agent

## Abstract

Liver fibrosis, characterized by excessive accumulation of the extracellular matrix (ECM), is caused by an imbalance between fibrogenesis and fibrolysis. Effective antifibrotic therapy requires the regulation of hepatic stellate cells (HSCs) through the production of collagen and matrix metalloproteinases (MMPs). The TGF-β/Smad and Wnt/β-catenin pathways play central roles in this regulation. In this study, we investigated whether IC-2, a Wnt/β-catenin inhibitor derived from ICG-001, reduces TGF-β/Smad and Wnt/β-catenin signaling in HSCs in vitro. We further examined the anti-fibrotic effects in two fibrosis models: carbon tetrachloride (CCl_4_)-treated mice and high-fat diet (HFD)-fed ob/ob mice. IC-2 markedly suppressed the TGF-β/Smad and Wnt/β-catenin pathway in HSCs. Furthermore, IC-2 markedly ameliorated liver fibrosis in CCl_4_-treated mice, accompanied with suppressed HSC activation and enhanced collagenolytic activity. IC-2 also attenuated fibrosis in the livers of HFD-fed mice. Co-immunoprecipitation analysis confirmed that IC-2 disrupts the β-catenin/CREB-binding protein (CBP) complex. IC-2 specifically induced MMP-1 expression in human HSCs, while the JNK inhibitor SP600125 suppressed IC-2-mediated MMP-1 induction at 48 h, but not at 24 h. It is suggested that this delayed effect may be due to a delayed transcriptional response or indirect signaling cascade downstream of JNK activation. These findings demonstrate that IC-2 mitigates liver fibrosis by inhibiting HSC activation and promoting collagenolysis, highlighting its therapeutic potential in cirrhosis.

## 1. Introduction

Cirrhosis is a major cause of morbidity and mortality regardless of its underlying etiology [1]. Thus, it imposes a substantial burden on healthcare systems worldwide. Cirrhosis accounted for approximately 1.48 million deaths in 2019 alone [2]. The U.S. Food and Drug Administration recently approved Rezdiffra (resmetirom) and Wegovy (semaglutide) for the treatment of metabolic dysfunction-associated steatohepatitis (MASH) and moderate-to-advanced liver fibrosis in adults. However, no targeted antifibrotic therapies have been approved for cirrhosis arising from other etiologies. This highlights the urgent need to develop effective antifibrotic agents for the clinical management of cirrhosis.

Fibrosis is a pathological condition characterized by the aberrant accumulation of the extracellular matrix (ECM) produced by myofibroblasts in response to chronic injury across various tissues and organs. In the liver, fibrosis is primarily driven by hepatic stellate cells (HSCs), which differentiate from their quiescent state into myofibroblasts [3]. Under physiological conditions, ECM turnover is tightly regulated by balanced production and degradation to maintain tissue homeostasis. However, this equilibrium is disrupted in chronic liver disease, leading to progressive liver fibrosis [4]. Therefore, effective therapeutic strategies may target the inhibition of fibrogenesis, the promotion of fibrolysis, or both.

In chronic liver injury, damaged hepatocytes and immune cells—including resident macrophages known as Kupffer cells and other inflammatory cell populations—contribute to liver fibrogenesis by releasing danger-associated molecular patterns (DAMPs), ROS, and pro-fibrogenic cytokines such as TNF-α, TGF-β1, and IL-1β [5,6]. TGF-β, a key mediator of HSC activation, promotes type I collagen expression through the Smad2/3-dependent signaling pathway [7]. Thus, targeting the TGF-β/Smad signaling axis is essential for the development of effective antifibrotic therapies. TGF-β receptor I phosphorylates Smad2/3 upon ligand binding, thereby enabling their translocation to the nucleus and subsequent activation of target genes such as α-smooth muscle actin (α-SMA) and collagen type I alpha 1 (*COL1A1*) [8]. Conversely, antagonists of the Wnt/β-catenin pathway can suppress TGF-β/Smad signaling [9]. β-catenin knockdown inhibited HSC activation in rat models [10], and Wnt antagonists ameliorated liver fibrosis in bile duct-ligated mice [11]. Therefore, the crosstalk between Wnt/β-catenin and TGF-β/Smad signaling has gained attention as a promising avenue for anti-fibrotic drug development [8,12,13,14].

CREB-binding protein (CBP) and E1A binding protein p300 (p300) function as co-activators and serve as transcriptional partners of β-catenin [15,16]. Furthermore, β-catenin and Smad4 form a complex with CBP on the α-SMA promoter to regulate α-SMA transcription [17]. Inhibitors that disrupt β-catenin/CBP complex formation, such as ICG-001 [18] and its derivative PRI-724, ameliorated liver fibrosis in various mouse models [19,20,21]. Thus, β-catenin/CBP interaction represents a promising therapeutic target for liver fibrosis.

In our previous study, we developed IC-2, a novel Wnt/β-catenin signaling inhibitor structurally derived from ICG-001 [22]. Unlike existing Wnt/β-catenin inhibitors, IC-2 not only effectively suppresses Wnt/β-catenin signaling in human mesenchymal stem cells (MSCs) but also induces the expression of key fibrolytic enzymes, including matrix metalloproteinase (MMP)-1, MMP-13, and MMP-14 [23,24]. Given its ability to inhibit fibrogenesis and promote fibrolysis, we evaluated the antifibrotic efficacy of IC-2 in two distinct liver fibrosis mouse models: carbon tetrachloride (CCl_4_)-induced hepatic fibrosis and metabolic dysfunction-associated steatohepatitis (MASH). Our findings demonstrate that IC-2 strongly suppresses liver fibrosis, suggesting that it is a promising inhibitor of liver fibrosis.

## 2. Results

### 2.1. IC-2 Reduces TGF-β/Smad and Wnt/β-Catenin Signaling in HSCs

As HSCs play a pivotal role in liver fibrosis, we first investigated the interaction between the Wnt/β-catenin and TGF-β/Smad pathways in HSCs. We performed siRNA-mediated knockdown to assess the effect of β-catenin depletion on HSC activation. Optimization of the siRNA concentration using a T-cell factor/lymphoid enhancer factor (TCF/LEF)-driven reporter assay revealed a marked reduction in promoter activity at 50–200 nM siRNA (Supplemental Figure S1A). β-catenin knockdown attenuated TGF-β/Smad signaling, as indicated by decreased p3TP-lux reporter activity (Supplemental Figure S1B). Phosphorylated Smad3 levels were consistently reduced following β-catenin knockdown (Supplemental Figure S1C). Moreover, β-catenin depletion in HSCs reduced the expression of α-SMA and type I collagen (Supplemental Figure S1D), which are regulated by phospho-Smad3. Collectively, these findings provide direct evidence of crosstalk between Wnt/β-catenin and TGF-β/Smad signaling in the regulation of human HSC activation.

### 2.2. IC-2 Inhibits HSC Activation by Blocking Both Wnt/β-Catenin and TGF-β Signaling

**Figure 1:**
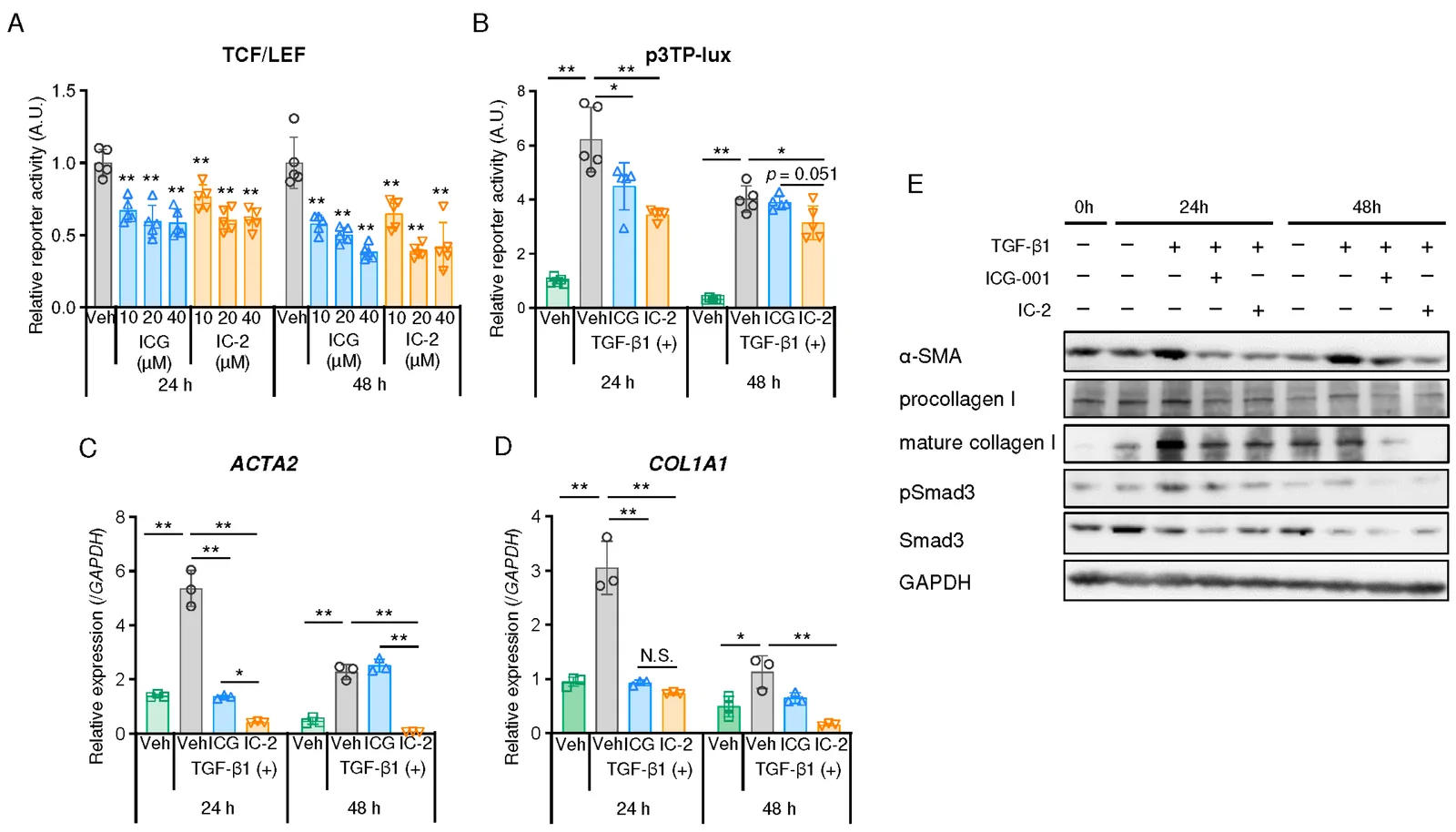
IC-2 and ICG-001 inhibit HSC activation by suppressing Wnt/β-catenin and TGF-β/Smad signaling. (**A**,**B**) TCF/LEF (**A**) and p3TP-lux (**B**) reporter gene assays in HSCs treated with IC-2 or ICG-001. (**C**,**D**) qPCR analysis of *ACTA2* (**C**) and *COL1A1* (**D**) mRNA expression in HSCs treated with IC-2 or ICG-001 in the presence or absence of 2.5 ng/mL TGF-β. (**E**) Representative Western blots for α-SMA, procollagen-I, mature collagen-I, p-Smad3, and total Smad3. Data are expressed as the mean ± SEM. Statistical significance was determined by ANOVA, followed by Tukey’s post-hoc test. * *p* < 0.05, ** *p* < 0.01 compared with the DMSO control; *n* = 5 (**A**,**B**) and *n* = 3 (**C**,**D**).

In our previous study, we successfully synthesized IC-2 (Supplemental Figure S2A), a derivative of ICG-001 (Supplemental Figure S2B), which effectively inhibits the Wnt/β-catenin signaling pathway in human MSCs [22]. In a subsequent study, we discovered that IC-2, unlike ICG-001, uniquely possesses a potent capacity to induce MMP-1 expression in human mesenchymal stem cells [23]. Based on these previous findings, we selected IC-2 to evaluate its dual therapeutic potential in suppressing fibrogenesis and actively restoring fibrolysis in liver fibrosis. In the present study, we explored the therapeutic potential of IC-2 against liver fibrosis. ICG-001, a promising anti-fibrotic candidate, was used as the reference compound [21,25,26]. We first evaluated the effects of IC-2 and ICG-001 on the Wnt/β-catenin pathway in human HSCs. Both compounds inhibited TCF and LEF activity at 24 h. At 48 h, 20 μM IC-2 reduced the reporter activity by approximately 60% of the baseline level (0 h), whereas 40 μM ICG-001 caused a comparable reduction (Figure 1A).

We used 20 μM IC-2 and ICG-001 in subsequent experiments, as they both strongly affected cell viability at 40 μM (Supplemental Figure S3). The p3TPlux reporter assay demonstrated that IC-2 substantially suppressed TGF-β/Smad signaling activated by TGF-β at 24 and 48 h (Figure 1B). In contrast, ICG-001 reduced p3TPlux activity at 24 h, but not at 48 h. IC-2 strongly suppressed TGF-β1-induced HSC activation, as indicated by reduced *ACTA2* and *COL1A1* expression at 24 and 48 h (Figure 1C,D). However, ICG-001 reduced *ACTA2* and *COL1A1* expression at 24 h, but not at 48 h. IC-2 consistently reduced α-SMA levels at 24 h and showed a more potent inhibitory effect than ICG-001 at 48 h (Figure 1E). Furthermore, IC-2 and ICG-001 reduced the expression of procollagen-I and mature collagen-I at 24 and 48 h. The inhibitory effects of IC-2 and ICG-001 on TGF-β/Smad signaling were further confirmed by reduced phospho-SMAD3 levels. These findings suggest that the inhibitory effect of IC-2 on human HSC activation results from its ability to block TGF-β/Smad signaling through inhibition of the Wnt/β-catenin pathway. In addition to human HSCs, we evaluated the effects of IC-2 on murine HSCs in vitro (Supplemental Figure S4). IC-2 markedly inhibited *Acta2* mRNA expression at 24 h and 48 h in primary murine HSCs, showing greater potency than that of ICG-001 (Supplemental Figure S4A). IC-2 also reduced *Col1a1* mRNA levels more effectively at both time points (Supplemental Figure S4B). Furthermore, IC-2 strongly reduced the expression of connective tissue growth factor (*Ccn2*) and platelet-derived growth factor-β (*Pdgfb*) compared to ICG-001 (Supplemental Figure S4C,D); these growth factors are critical mediators in HSC activation. Collectively, these results suggest that IC-2 inhibits HSC activation and fibrosis more effectively than ICG-001 in both human and murine HSCs.

### 2.3. IC-2 Suppressed HSC Activation and Fibrosis in CCl_4_-Treated Mice

**Figure 2:**
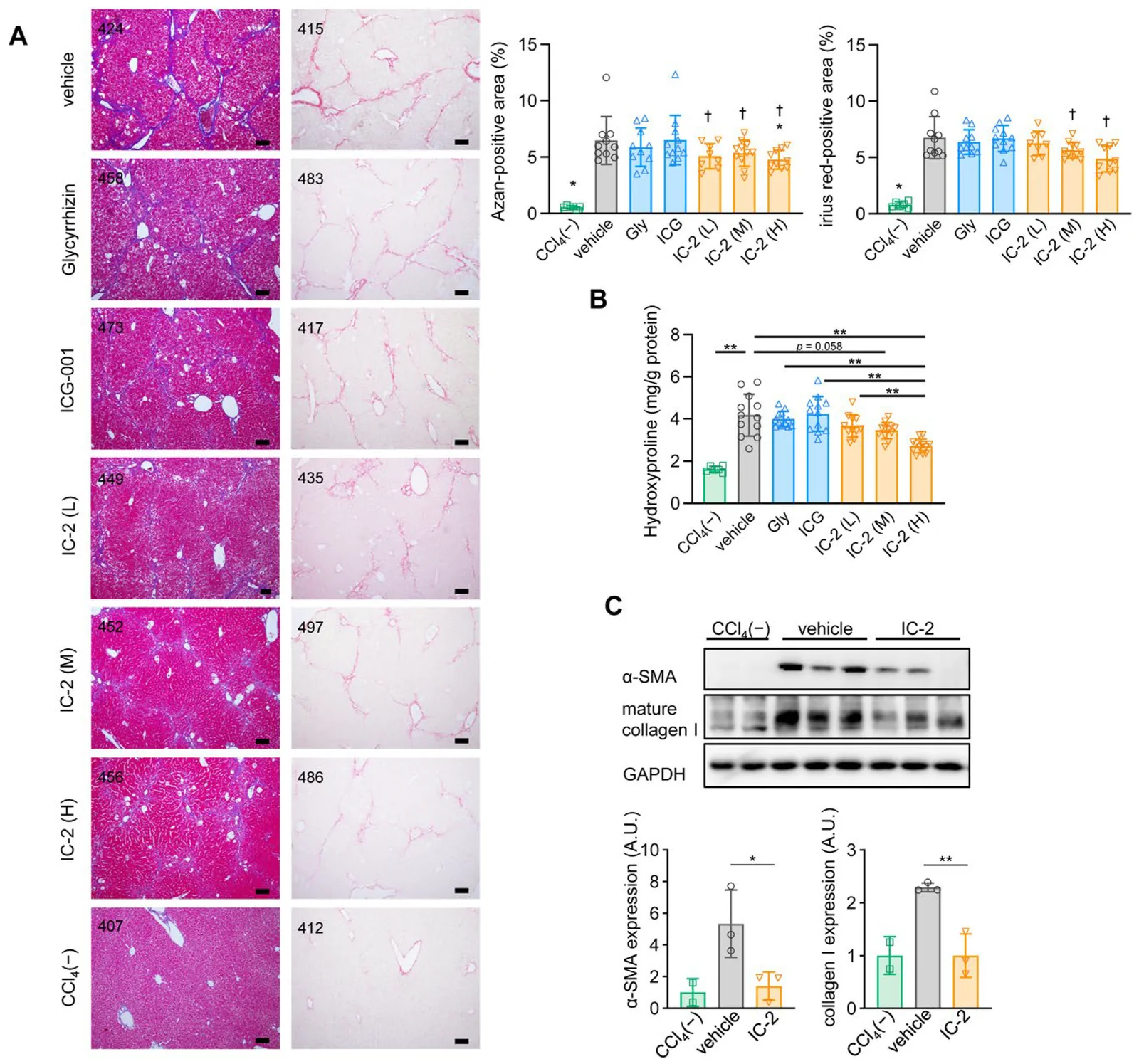
IC-2 attenuates liver fibrosis in a CCl_4_-induced mouse model. (**A**) Representative images of Azan and Sirius red staining of liver sections (**left**) and subsequent quantification of the positive areas (**right**). Scale bar: 100 µm. (**B**) Hydroxyproline content in liver tissues across the specified groups. Western blot analysis and densitometric quantification of α-SMA and type I collagen expression in liver tissues. Three independent blots were assessed utilizing Image J software (version 1.53i) and expressed as arbitrary units. Data are expressed as the mean ± SEM. Each dot represents an individual animal. For (**A**,**B**), *n* = 10–12 except for CCl_4_(−) (*n* = 6). For (**C**), three independent blots were quantified (*n* = 3 per group, except for CCl_4_(−), *n* = 2). Statistical significance was determined by Steel’s test (* in (**A**)) or Williams’ test († in (**A**)) for non-parametric and trend comparisons, respectively; Tukey’s test (**B**); or Student’s *t*-test (**C**). * *p* < 0.05, ** *p* < 0.01 vs. vehicle; † *p* < 0.05 vs. vehicle.

The antifibrotic effect of IC-2 was examined using a CCl_4_-induced liver fibrosis model (Supplemental Figure S5). Glycyrrhizin alleviates CCl_4_-induced liver fibrosis via Smad3 inhibition [27]. Therefore, it was used as a positive control. After 4 weeks of CCl_4_ administration, mice were administered glycyrrhizin, ICG-001, or one of three doses of IC-2 (5.3, 10.6, and 21.2 mg/kg) concurrently with continued CCl_4_ treatment. The 10.6 mg/kg dose of IC-2 corresponds to 20 μM IC-2, the effective concentration that inhibits HSC activation in vitro. No significant differences in body weight, liver weight, or liver-to-body weight ratio were observed among the groups (Supplemental Table S1). Similarly, serum AST and ALT levels were comparable across groups. Although some groups exhibited slightly higher total bilirubin levels than the vehicle group, all the values remained within the normal range. Histological analysis revealed a significant reduction in liver fibrosis in mice treated with 21.2 mg/kg IC-2, as assessed by Azan staining using the Steel test (Figure 2A). The William test also revealed significant reductions in the 5.3 mg/kg, 10.6 mg/kg, and 21.2 mg/kg groups, indicating the dose-dependent effects of IC-2 (Figure 2A). Sirius red staining further demonstrated that IC-2 at 10.6 and 21.2 mg/kg effectively reduced collagen deposition compared to the vehicle (Figure 2A). Hepatic hydroxyproline content decreased in a dose-dependent manner (Figure 2B). Furthermore, CCl_4_-induced HSC activation was attenuated in mice treated with 21.2 mg/kg IC-2, as indicated by reduced α-SMA and type-I collagen expression during Western blotting (Figure 2C). Collectively, these findings indicate that IC-2 effectively suppressed HSC activation and liver fibrosis in vivo.

### 2.4. Effects of IC-2 on Metabolic Dysfunction-Associated Steatohepatitis in ob/ob Mice

**Figure 3:**
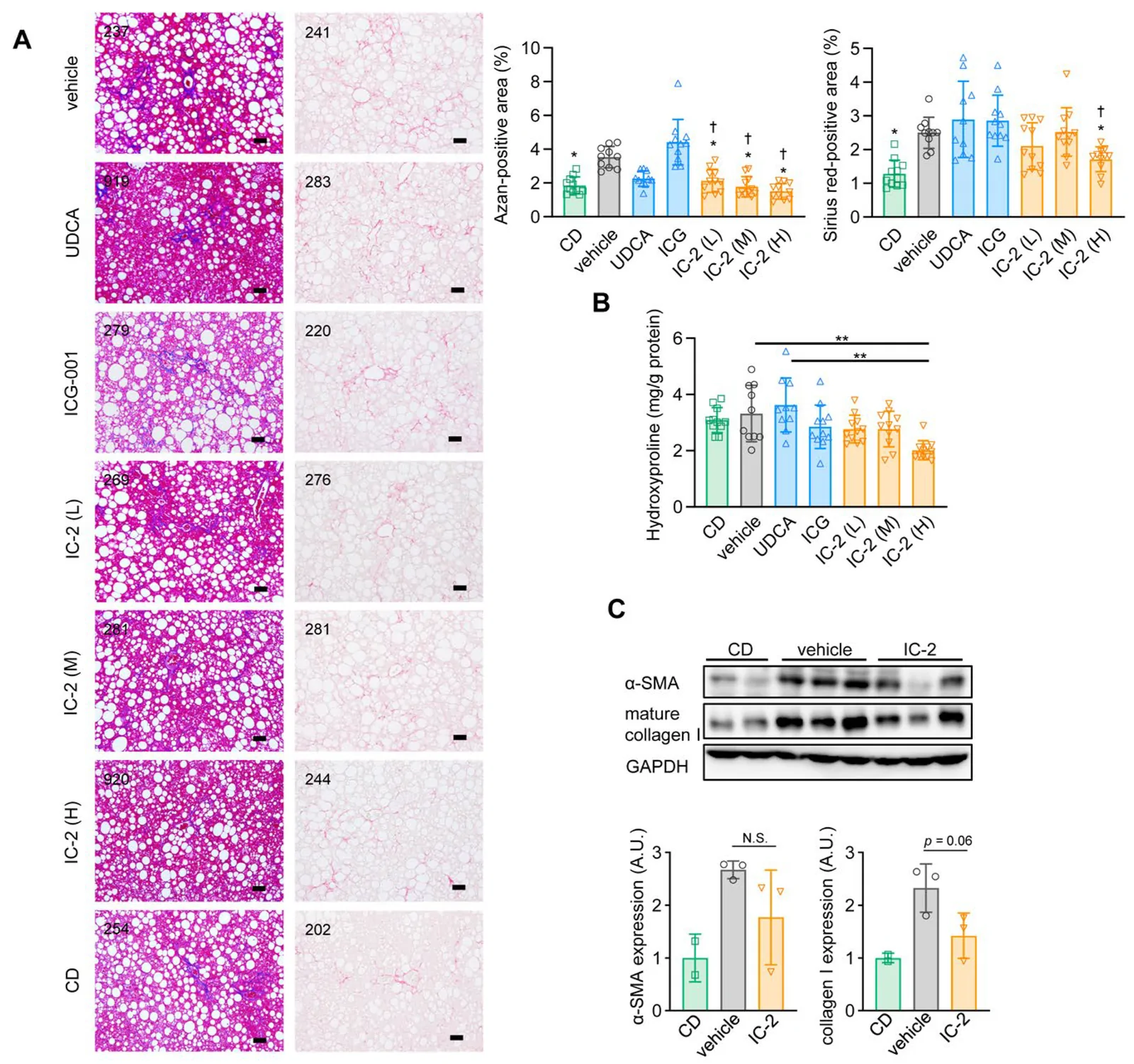
The anti-fibrotic effect of IC-2 in high-fat-diet-fed mice. (**A**) Representative images of Azan and Sirius red staining of liver sections (**left**) and quantification of the positive areas (**right**). Scale bar: 50 µm. (**B**) Hydroxyproline content in liver tissues across the specified groups. (**C**) Western blot analysis and densitometric quantification of α-SMA and type I collagen expression in liver tissues. Data are expressed as the mean ± SEM. Each dot represents an individual animal. For (**A**,**B**), *n* = 10–12 per group. For (**C**), two independent blots were quantified using ImageJ version 1.53i (*n* = 3 per group, except for the control, *n* = 2). Statistical significance was determined by Steel’s test (* in (**A**)) or Williams’ test († in (**A**)); Tukey’s test (**B**); or Student’s *t*-test (**C**). * *p* < 0.05, ** *p* < 0.01 vs. the vehicle; † *p* < 0.05 vs. the vehicle.

To evaluate the effects of IC-2 on liver fibrosis arising from different pathophysiological mechanisms, we evaluated its therapeutic efficacy in a murine MASH model. The ob/ob mice were fed a high-fat diet (HFD) for 12 weeks, and therapeutic interventions were administered during the final 6 weeks (Supplemental Figure S5). Ursodeoxycholic acid (UDCA) was used as a positive control. Liver weight, liver-to-body weight ratio, ALT levels, and blood glucose levels significantly decreased in the UDCA group (Supplemental Table S2). Azan staining revealed that the Azan-positive area was attenuated in all three IC-2 treatment groups, as indicated by both the Steel and Williams tests (Figure 3A). In contrast, Sirius red staining showed a significant reduction only in the 21.2 mg/kg IC-2 group (Figure 3A). Neither UDCA nor ICG-001 significantly alleviated hepatic fibrosis. Hepatic hydroxyproline content decreased in the 21.2 mg/kg IC-2 group (Figure 3B). Although α-SMA and type I collagen expression levels exhibited a downward trend relative to the vehicle group, quantitative analyses of signal intensities did not show statistically significant differences (Figure 3C). Taken together, these findings suggest that IC-2 attenuates liver fibrosis in the MASH mouse model.

### 2.5. IC-2 Elicits an Inhibitory Effect on Profibrotic Actions and Enhances Fibrolytic Responses In Vitro

**Figure 4:**
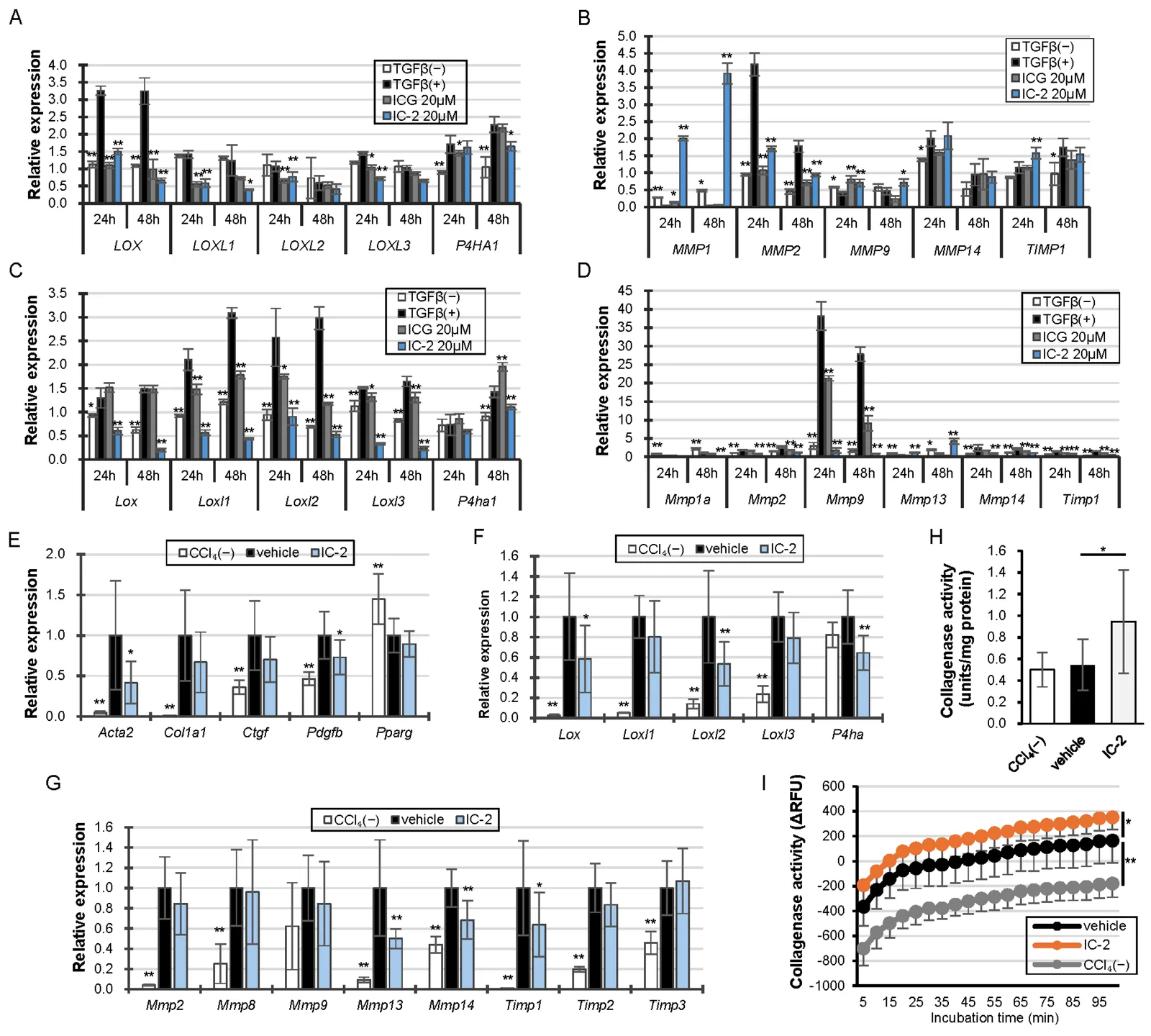
IC-2 modulates profibrogenic and fibrolytic gene expression in vitro and in vivo. (**A**–**D**) qPCR analysis of profibrogenic (**A**,**C**) and fibrolytic (**B**,**D**) mRNA expression in human (**A**,**B**) and murine primary cells (**C**,**D**) treated with IC-2 or ICG-001 in the presence of TGF-β (2.5 ng/mL for human cells; 10 ng/mL for murine cells) for 24 or 48 h. (**E**–**G**) qPCR analysis of specific profibrogenic and fibrolytic genes in liver tissues from mice with CCl_4_-induced fibrosis treated with IC-2 (21.2 mg/kg). (**H**,**I**) Collagenase activity in liver tissues from CCl_4_-treated mice, as assessed by Type I collagenase (**H**) and collagen degradation/zymography (**I**) assays. Data are expressed as the mean ± SEM. *GAPDH*/*Gapdh* was used as the internal control. Statistical significance was determined using Dunnett’s test (**A**–**G**), the Kruskal–Wallis test (**H**), or two-way ANOVA followed by Tukey’s post-hoc test (I). * *p* < 0.05, ** *p* < 0.01 compared with the TGF-β(+)/DMSO control or vehicle-treated CCl_4_ group. For (**A**–**D**), *n* = 3 per group; for (**E**–**G**), *n* = 6 (CCl_4_(−)), *n* = 10 (vehicle), and *n* = 12 (IC-2); for (**H**,**I**), *n* = 12 per group, except for CCl_4_(−) (*n* = 6).

To elucidate the mechanisms underlying the anti-fibrotic effects of IC-2, we investigated the expression profiles of pro-fibrogenic and pro-fibrolytic genes in vitro using human LX-2 (Figure 4A,B) and murine primary (Figure 4C,D) HSCs. Under TGF-β-induced activation, IC-2 significantly attenuated the mRNA expression of lysyl oxidase (*LOX*) at both 24 and 48 h in human HSCs. The expression levels of lysyl oxidase-like 1 (*LOXL1*) and lysyl oxidase-like 3 (*LOXL3*) also decreased. This inhibitory effect was more pronounced in murine HSCs, in which the expression levels of *Lox*, *Loxl1*, *Loxl2*, and *Loxl3* were substantially reduced (Figure 4C). IC-2 exhibited a more sustained and potent inhibitory effect at 48 h than ICG-001, which showed similar effects to IC-2 in human HSCs at 24 h. This superiority was even more evident in murine HSCs, where IC-2 produced a markedly stronger reduction in pro-fibrogenic gene expression than ICG-001 at both 24 and 48 h. Under steady-state conditions without TGF-β stimulation, IC-2 treatment for 48 h markedly inhibited *LOX*, *LOXL1*, and *LOXL2* expression in human HSCs (Supplemental Figure S6A). In contrast, IC-2 markedly reduced the expression of all analyzed pro-fibrogenic genes in murine HSCs (Supplemental Figure S6C).

We subsequently focused on several fibrolytic enzymes involved in type I collagen degradation, which constitute 60–70% of the ECM proteins in cirrhotic liver tissues [28]. Under TGF-β-induced HSC activation, IC-2 treatment significantly increased *MMP1* expression in human HSCs, whereas ICG-001 did not alter the expression of this gene (Figure 4B). Although the expression of *Mmp1a* was not upregulated in murine HSCs, that of *Mmp13*, a functional murine homolog of human *MMP1* [29,30], was markedly elevated after IC-2 treatment (Figure 4D). In contrast, *Mmp2* and *Mmp9* expression decreased at both 24 and 48 h in murine HSCs. Under steady-state conditions without TGF-β stimulation, *MMP1* and *Mmp13* expression also increased at 24 and 48 h in human and murine HSCs, respectively (Supplemental Figure S6B,D). These findings indicate that IC-2 suppresses the expression of pro-fibrogenic genes while promoting that of fibrinolytic genes in vitro.

### 2.6. IC-2 Promotes Fibrolysis and Suppresses Pro-Fibrogenic Activity by Inhibiting HSC Activation In Vivo

We further validated anti-fibrogenic and fibrolytic effects in vivo using a CCl_4_-induced liver fibrosis mouse model. Consistent with the in vitro findings (Figure 1 and Supplemental Figure S4), 21.2 mg/kg IC-2 suppressed *Acta2* expression (Figure 4E). Furthermore, the expression of all pro-fibrogenic genes exhibited a downward trend, with significant reductions in *Lox*, *Loxl2*, and prolyl 4-hydroxylase subunit alpha (*P4ha*) levels (Figure 4F). Further analyses of ECM-related gene expression revealed no substantial changes in MMPs. However, tissue inhibitor of metalloproteinases 1 (*Timp1)* expression decreased following IC-2 treatment (Figure 4G). In contrast, type I collagenase activity significantly increased in the liver tissues of mice treated with 21.2 mg/kg IC-2 (Figure 4H). This finding was further supported by an independent commercial collagen degradation assay (Figure 4I). Although hepatic *Mmp13* mRNA levels were not elevated in IC-2-treated mice (Figure 4G), both functional assays revealed a pronounced increase in collagenolytic activity after IC-2 administration (Figure 4H,I). These results suggest that IC-2 promotes fibrolysis by reducing *Timp1* expression or facilitating localized enzymatic activation within the fibrotic microenvironment. Collectively, these findings indicate that IC-2 effectively promoted fibrolysis and accelerated the resolution of established fibrosis in the CCl_4_-induced liver fibrosis mouse model.

### 2.7. Mechanisms Underlying the Regulation of Wnt/β-Catenin Signaling and MMP-1 Expression by IC-2

**Figure 5:**
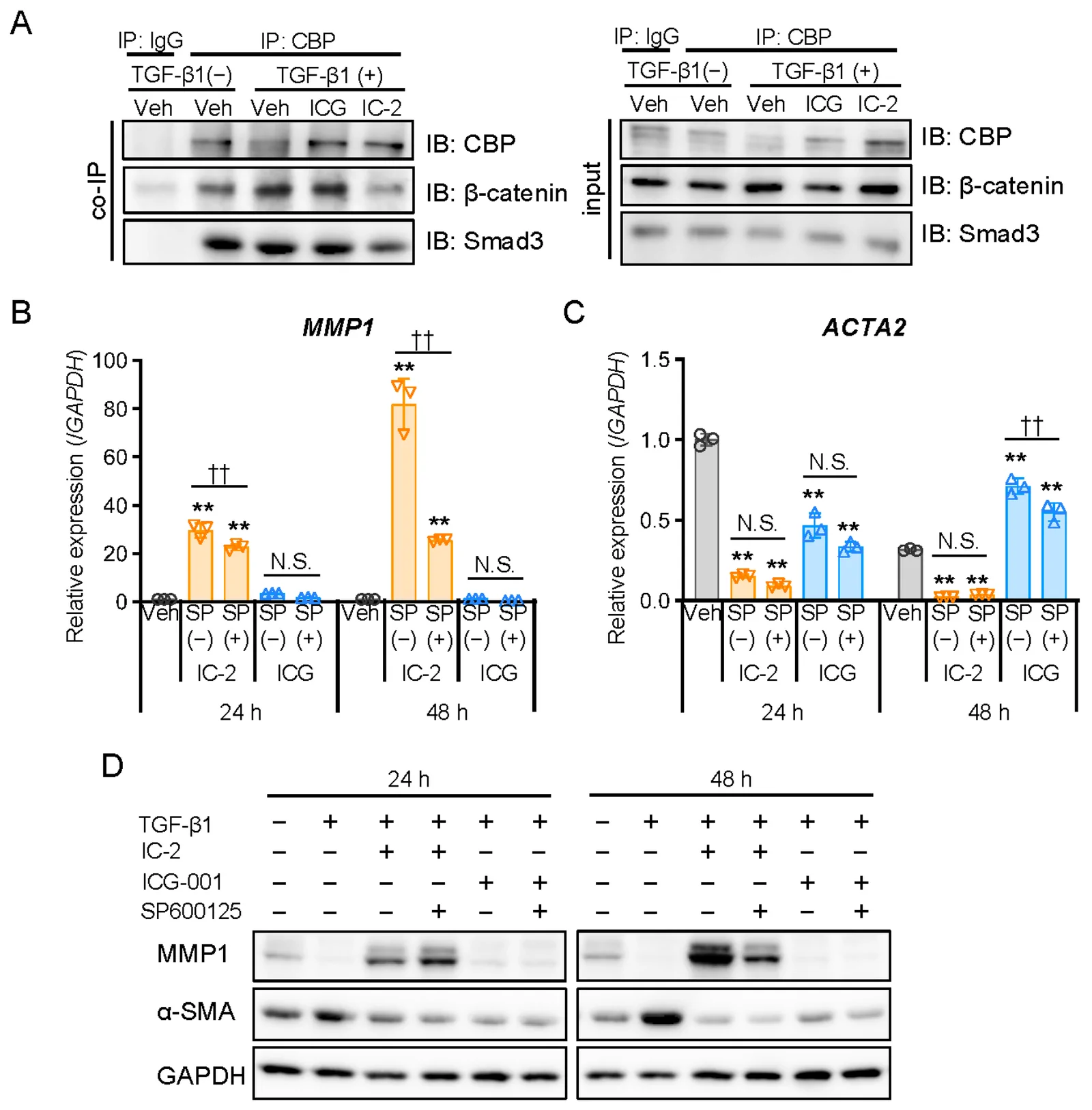
IC-2 inhibits β-catenin/CBP interaction and induces *MMP1* expression via JNK signaling. (**A**) Co-IP of β-catenin and CBP from nuclear extracts. Input (5%) is shown in the left panel. (**B**,**C**) qPCR analysis of *MMP1* (**B**) and *ACTA2* (**C**) mRNA expression in LX-2 cells treated with 2.5 ng/mL TGF-β and IC-2 or ICG-001 in the presence or absence of the JNK inhibitor SP600125 (10 µM). (**D**) Representative Western blots of MMP-1 and α-SMA expression in LX-2 cells following the indicated treatments. Data are expressed as the mean ± SEM of three independent experiments (*n* = 3). Statistical significance was determined by ANOVA followed by Tukey’s post-hoc test. ** *p* < 0.01 compared with TGF-β/DMSO; †† *p* < 0.01 compared with IC-2 without SP600125.

To elucidate the IC-2 mechanism of action, we first investigated whether it interferes with the interaction between β-catenin and CBP. ICG-001 inhibited the formation of the CBP/β-catenin complex without altering the expression levels of either protein in a previous study [18]. In the present study, co-IP analyses of nuclear extracts revealed that IC-2 disrupted β-catenin/CBP complex formation (Figure 5A). Moreover, IC-2 appeared to be a more potent inhibitor than ICG-001. Collectively, these findings suggest that IC-2 suppresses the Wnt/β-catenin signaling pathway by inhibiting β-catenin/CBP complex formation.

In addition to inhibiting the Wnt/β-catenin pathway, IC-2 upregulated *MMP1* and *Mmp13* expression in human and murine HSCs, respectively. As the JNK pathway regulates *MMP1* expression [31,32], we investigated whether JNK activation contributes to the IC-2-induced upregulation of *MMP1* expression. *MMP1* expression increased in response to IC-2, but not to ICG-001. However, the JNK inhibitor SP600125 effectively suppressed this increase (Figure 5B) at 48 h, but not at 24 h. These data suggest that IC-2-mediated *MMP1* induction may be due to the activation of the JNK pathway; this delayed response suggests that JNK activation mediates a delayed transcriptional response or an indirect signaling cascade regulating MMP-1 expression. In addition, no differences in *ACTA2* expression were observed between SP600125-treated and untreated cells in the IC-2-treated group. This suggests that the IC-2-mediated suppression of *ACTA2* occurred independently of JNK activation (Figure 5C). Western blotting confirmed these observations (Figure 5D). Taken together, these results demonstrate that IC-2 is a potent antifibrotic agent that exerts dual effects: suppression of HSC activation and induction of MMP-1 expression.

## 3. Discussion

Liver fibrosis is a complex and progressive condition that severely impairs hepatic function and accelerates the development of hepatocellular carcinoma (HCC). Despite its clinical significance, no approved therapy is available for this condition. TGF-β/Smad signaling is a central pathway driving HSC activation and the progression of liver fibrosis [7]. The present study elucidates the regulatory crosstalk between Wnt/β-catenin and TGF-β/Smad pathways during HSC activation. IC-2, a Wnt/β-catenin signal inhibitor, suppressed liver fibrosis by simultaneously inhibiting TGF-β/Smad signaling and suppressing Wnt/β-catenin signaling. These findings suggest that IC-2 is a compelling candidate for therapeutic intervention in liver fibrosis. In the present study, 5 mg/kg ICG-001, which was effective against liver fibrosis in a previous study [25], failed to suppress CCl_4_- or HFD-induced liver fibrosis. In contrast, 5.3 mg/kg IC-2, a dose similar to that of ICG-001, markedly reduced the Azan-positive area in CCl_4_- and HFD-induced liver fibrosis. The higher anti-fibrotic efficacy of IC-2 than that of ICG-001 in vivo was further supported by its pronounced inhibitory effects on HSC activation, as demonstrated in vitro (Supplemental Figure S4).

Dysregulation of Wnt/β-catenin signaling often leads to various serious diseases. Therefore, the development of small-molecule compounds that target the Wnt signaling pathway is critical [33]. Several CBP inhibitors that function as antagonists of the interaction between β-catenin and CBP have been developed [34]. ICG-001, along with PRI-724, C-82, and E-7386, belongs to this class of selective CBP/β-catenin signaling inhibitors [35]. ICG-001 inhibited CCl_4_-induced liver fibrosis through suppression of stromal CXCL12 [25], whereas PRI-724 suppressed HCV-induced liver fibrosis by attenuating α-SMA expression and inducing *Mmp8* expression [20]. Phase I clinical trials have demonstrated the safety of PRI-724; significant improvements in liver stiffness were reported [36]. Because IC-2 also disrupts the β-catenin/CBP interaction, it is categorized within the same class of inhibitors. Despite this shared mechanism, differences in their broader molecular interactions likely account for their distinct biological effects.

Importantly, our study revealed that IC-2 not only inhibits HSC activation but also significantly promotes the expression of MMP-1, an enzyme that is essential for the degradation of type I, II, and III collagens [37]. In human LX-2 cells, the induction of MMP-1 expression was uniquely observed following IC-2 treatment, whereas ICG-001 had no inductive effect on this fibrolytic enzyme (Figure 4B and Supplemental Figure S6B). Adenoviral delivery of *MMP1* into rats demonstrated a therapeutic effect against thioacetamide-induced liver fibrosis, indicating that MMP-1 effectively mitigated liver fibrosis [38]. In addition, the clinical relevance of MMP-1 in liver diseases has been increasingly recognized. Köylü et al. [39] recently demonstrated that the progression of fibrosis in MASH is associated with a decrease in serum MMP-1 levels. This clinical observation suggests that deficiency in MMP-1-mediated fibrolysis contributes to the irreversible accumulation of the ECM in advanced liver disease. Therefore, the ability of IC-2 to simultaneously suppress fibrogenesis and actively restore MMP-1 expression represents a considerable pharmacological advantage over first-generation CBP/β-catenin antagonists such as ICG-001.

Wnt/β-catenin signaling inhibited MMP expression in human articular chondrocytes. The subsequent knockdown of β-catenin increased MMP-1 and MMP-13 expression independent of TCF-4 and LEF-1 [40]. In our recent study, Dkk-1, a soluble inhibitor of Wnt/β-catenin signaling, decreased *ACTA2* and increased *MMP1* mRNA expression in human LX-2 HSCs [41]. As the IC-2-induced increase in *MMP1* expression was considerably strong (Figure 4B), alternative pathways likely contributed to this potent action. Consistent with this finding, the JNK inhibitor SP600125 effectively inhibited the IC-2-mediated increase in *MMP1* expression, whereas *ACTA2* suppression remained unaffected (Figure 5B–D). These results indicate that IC-2 activates a JNK-dependent fibrolytic pathway. This was distinct from its established role in the inhibition of HSC activation through β-catenin/CBP disruption. This unique dual-action mechanism distinguishes IC-2 from conventional CBP/β-catenin antagonists such as ICG-001, highlighting its potential as a superior therapeutic candidate for advanced liver cirrhosis. Based on this JNK-dependent mechanism, it is noteworthy that while IC-2 markedly induced *MMP1* in human HSCs, it upregulated *Mmp13* in murine HSCs. Rodents lack a direct structural ortholog of human *MMP1*, and *Mmp13* serves as the primary functional collagenase responsible for type I collagen degradation in mice [29,30]. To explore potential transcriptional mechanisms underlying this species-dependent expression, we conducted an in silico promoter analysis using JASPAR [42] across 3-kb promoter regions of human *MMP1*, human *MMP13*, murine *Mmp13*, and murine *Mmp1a*. Interestingly, composite binding motifs for JNK-responsive bZIP factors and Ets-family factors, such as ATF3::GABPA (UN1167.1), were uniquely shared between human *MMP1* and murine *Mmp13*, but were absent in human *MMP13* and murine *Mmp1a* (Table S2). Given that bZIP and Ets-family transcription factors can function downstream of JNK signaling, these conserved composite motifs might provide a plausible explanation for the species-appropriate induction of functional collagenases (human *MMP1* vs. murine *Mmp13*) by IC-2, although further experimental validation is required.

Furthermore, the Wnt/β-catenin signaling cascade facilitates tumor progression, metastasis, and resistance to pharmacological interventions [43,44]. We previously reported that IC-2 inhibits the proliferation of cancer stem cells [45,46]. As liver fibrosis is a precursor lesion to HCC, its suppression is expected to prevent HCC development in cirrhotic livers [47]. Despite these multifaceted therapeutic advantages, effective in vitro responses of IC-2 required micromolar concentrations, although it demonstrated robust antifibrotic efficacy in vivo without overt toxicity or hepatic dysfunction (Supplemental Tables S1 and S2). High working concentrations and potential narrow therapeutic windows represent a common hurdle for small-molecule CBP/β-catenin antagonists. The present study demonstrate that IC-2 represents a promising lead compound. Future efforts should focus on structural optimization with higher potency at lower doses, as well as optimizing drug delivery strategies to enhance therapeutic windows and facilitate its clinical application.

In summary, the results of our study support the hypothesis that IC-2 serves as a superior antifibrotic agent by targeting both fibrogenesis and fibrolysis. This innovative approach could provide a basis for future clinical applications and significantly improve clinical outcomes in patients with liver fibrosis.

## 4. Methods

### 4.1. Sex as a Biological Variable

Our study examined male mice exclusively due to the sex-dimorphic nature of liver fibrosis and cirrhosis. In humans, males exhibit a significantly higher incidence and faster progression of liver fibrosis, whereas females are relatively protected, primarily due to the anti-fibrotic and antioxidant roles of estrogen [48,49,50]. Consistent with these clinical observations, male mice display more severe disease phenotypes in both CCl_4_-induced and diet-induced fibrosis models [51,52,53]. Therefore, male mice were utilized to ensure a robust and reproducible fibro-inflammatory response. Further research is warranted to determine the applicability of these findings to female mice.

### 4.2. Cell Lines, Culture, and Reagents

LX-2 human HSCs were maintained in DMEM (Nissui Pharmaceutical Co., Ltd., Tokyo, Japan) supplemented with 1% FBS (Sigma–Aldrich, St. Louis, MO, USA), 4 mM L-glutamine, and 3500 mg/L glucose in a humidified environment with 5% CO_2_ at 37 °C. LX-2 cells were inoculated at 1.4 × 10^4^ cells/cm^2^ and exposed to recombinant TGF-β1 (2.5 ng/mL; R&D Systems, Inc., Minneapolis, MN, USA), IC-2, or ICG-001 at varying concentrations for 24 and 48 h. Mouse HSCs were acquired from Cell Applications, Inc. (San Diego, CA, USA) and cultured in mouse HSC growth medium (Cell Applications, Inc.) according to the manufacturer’s instructions. Mouse HSCs were seeded at 2.4 × 10^4^ cells/cm^2^ and treated with 10 ng/mL recombinant TGF-β1, IC-2, or ICG-001 of varying concentrations for 24 and 48 h.

The cells were cultured under the aforementioned conditions and subsequently used in all experimental procedures. IC-2 was synthesized in-house [22]. ICG-001 was purchased from Adooq Bioscience LLC. (Irvine, CA, USA). IC-2 and ICG-001 were dissolved in 0.1% DMSO (NACALAI TESQUE, Inc., Kyoto, Japan). SP600125 was purchased from FUJIFILM Wako Pure Chemical Co. (Osaka, Japan).

### 4.3. CCl_4_-Induced Liver Fibrosis Model

Mice were maintained in sterilized cages in a controlled environment with a 12-h light/dark cycle at 22 ± 2 °C and 50–60% humidity. Seven-week-old male C57BL/6J mice were obtained from CLEA (Tokyo, Japan). Mice were intraperitoneally administered CCl_4_ diluted in corn oil (1:9 *v*/*v*) (FUJIFILM Wako Pure Chemical Corp.) at a dosage of 0.2 mL/kg BW thrice a week for four weeks (Figure 3A). Mice were then stratified into six groups based on biochemical parameters such as serum ALT and AST levels, total bilirubin levels, and BW. The designated experimental groups were as follows: (1) CCl_4_ + vehicle, (2) CCl_4_ + glycyrrhizin (165 mg/kg), (3) CCl_4_ + ICG-001 (5 mg/kg; Selleck Biotechnology, Yokohama, Japan), (4) CCl_4_ + IC-2 (5.3 mg/kg), (5) CCl_4_ + IC-2 (10.6 mg/kg), and (6) CCl_4_ + IC-2 (21.2 mg/kg). After grouping, each drug or vehicle was intraperitoneally administered three times a week for four weeks, starting the day after CCl_4_ administration. IC-2 or ICG-001 was delivered in a vehicle containing 10% ethanol, 5% Cremophor EL (Nacalai Tesque), and 85% D5W (dextrose 5% in water). Glycyrrhizin (Tokyo Chemical Industry Co., Ltd., Tokyo, Japan) was dissolved in saline, and the pH was adjusted to neutral. On the day after the final administration of each therapeutic agent, mice were deeply anesthetized with a combination of medetomidine (0.3 mg/kg), midazolam (4.0 mg/kg), and butorphanol (5.0 mg/kg) via intraperitoneal injection to minimize pain and distress, and were humanely euthanized by complete blood collection via the inferior ve-na cava before tissue harvesting. Blood samples were used for biochemical analyses. The left lateral lobe of each liver was fixed in 4% paraformaldehyde for histological evaluation. The right median lobe and remaining liver tissues were snap-frozen in liquid nitrogen and stored at −80 °C for additional analyses.

### 4.4. Establishment of the HFD-Fed ob/ob Mouse Model

Seven-week-old male C57BL/6J Ham Slc-*ob/ob* mice were obtained from Japan SLC Inc. (Shizuoka, Japan). To induce MASH, mice were fed an HFD containing 40% kcal derived from fat, 22% (*w*/*w*) fructose, and 2% (*w*/*w*) cholesterol (D09100301; Research Diets, Inc., New Brunswick, NJ, USA) [54]. The control diet was D09100304 (Research Diets, Inc.). After six weeks of the HFD regimen, the mice were divided into six groups with comparable average BW and biochemical parameters. The experimental groups were identical to those used in the CCl_4_-induced liver fibrosis model, except for group 2, which was orally administered ursodeoxycholic acid (UDCA, 150 mg/kg; Mitsubishi Tanabe Pharma, Osaka, Japan) in saline. Each pharmacological agent was administered for an additional six weeks together with the HFD.

### 4.5. Reporter Assay

To investigate Wnt/β-catenin signaling reporter activity, LX-2 cells were transduced with a lentiviral vector expressing TCF-4 motif reporter constructs (Cignal Lenti TCF/LEF Reporter [luc]; QIAGEN N.V., Venlo, The Netherlands), together with a control Renilla lentivirus (Cignal Lenti TK Renilla Control [luc]; Qiagen N.V.). The siRNA targeting β-catenin was purchased from Hokkaido System Science Co., Ltd. (Hokkaido, Japan). The sequence was as follows: sense strand, 5′-GGUGGUGGUUAAUAAGGCUtt-3′; antisense strand, 3′-gtCCACCACCAAUUAUUCCGA-5′ [55]. siRNA was transfected with the Lipofectamine 2000 reagent (Thermo Fisher Scientific Inc., Waltham, MA, USA) at 24 h after virus transduction. ICG-001 or IC-2 was administered 24 h after viral transduction.

To evaluate the activation of the TGF-β/Smad signaling pathway, LX-2 cells were co-transfected with a TGF-β responsive luciferase reporter plasmid (p3TP-Lux; Addgene, Watertown, MA, USA) and a control pRL-TK vector (Promega, Madison, WI, USA) using Lipofectamine 2000. To evaluate the effect of β-catenin siRNA, cells were co-transfected with siRNA and reporter plasmids, followed by treatment with recombinant human TGF-β1 at 6 h after transfection. LX-2 cells were subsequently treated with TGF-β and/or ICG-001 or IC-2 6 h after transfection with reporter plasmids. Firefly and Renilla luciferase activities were measured using the dual-luciferase reporter assay system (Promega), and firefly luciferase activity was normalized to Renilla luciferase activity.

### 4.6. RNA Extraction and Reverse Transcription Quantitative Real-Time PCR

Total RNA was isolated from cultured cells or hepatic tissues using the RNeasy Plus Mini Kit (QIAGEN N.V.) according to the manufacturer’s instructions. RNA was reverse-transcribed using SuperScript II Reverse Transcriptase (Thermo Fisher Scientific) with oligo (dT)_18_ primers according to the manufacturer’s instructions. Quantitative PCR (qPCR) was performed using the ViiA7 Real-Time PCR platform (Thermo Fisher Scientific) with THUNDERBIRD SYBR qPCR Mix (TOYOBO Co., Ltd., Osaka, Japan). The primers specific for the genes of interest are listed in Table S1.

### 4.7. Western Blotting

Cells and frozen liver tissues were lysed in modified RIPA buffer (50 mM Tris-HCl, pH 7.4; 150 mM NaCl; 0.25% sodium deoxycholate; 0.1% SDS; 1% NP-40; 1 mM EDTA; and 5% glycerol) supplemented with cOmplete protease inhibitor and PhosSTOP (Sigma–Aldrich). After sonication at 200 W for 30 s, the homogenate was centrifuged to collect the supernatant. The primary antibodies used were: anti-β-catenin (1:1000; sc-7963, Santa Cruz Biotechnology, Dallas, TX, USA), anti-α-SMA (1:500; M0851, Agilent Technologies, CA, USA), anti-Col1a1 (1:500; sc-293182, Santa Cruz Biotechnology), anti-Smad3 (1:500; 9523S, Cell signaling Technology, Danvers, MA, USA), anti-phospho Smad3 (1:500; 9520S, Cell signaling Technology), anti-MMP1 (1:1000; 10371-2-AP, Proteintech Japan, Tokyo, Japan), anti-GAPDH (1:2000; MAB374, Merck KGaA, Darmstadt, Germany), and anti-β-actin (1:5000; sc-1616, Santa Cruz Biotechnology).

### 4.8. Co-IP Assay

LX-2 cells were treated with TGF-β1 and/or ICG-001 or IC-2. Twenty-four hours post-treatment, the cytoplasmic fraction was separated utilizing a hypotonic buffer (10 mM HEPES, pH 7.9; 1.5 mM MgCl_2_; 10 mM KCl; 0.1 mM EDTA; 0.1% NP-40; 1 mM DTT; and cOmplete protease inhibitor), whereas the nuclear fraction was extracted from the supernatant following centrifugation at 20,000× *g* for 5 min with a nuclear extraction buffer (20 mM HEPES, pH 7.9; 1.5 mM MgCl_2_; 400 mM NaCl; 0.1 mM EDTA; 0.1% NP-40; 10% glycerol; 1 mM DTT, and the cOmplete protease inhibitor). A total of 150 μg of nuclear protein was solubilized in 1 mL IP buffer (20 mM HEPES, pH 7.5; 150 mM NaCl; 1 mM EDTA; 0.1% NP-40; 1 mM DTT; and the cOmplete protease inhibitor). Before IP, lysates were pre-cleaned with 20 μL Protein A/G Agarose beads (Santa Cruz Biotechnology) for 30 min. Co-IP was conducted overnight with 2 μg of mouse normal IgG (sc-3877; Santa Cruz Biotechnology) or anti-CBP antibody (sc-7300; Santa Cruz Biotechnology). The immuno-complex was retrieved through incubation with 50 μL of Protein A/G agarose beads (50% Slurry) for 1 h. After five washes with IP buffer, the immuno-complex was eluted through incubation with 50 μL of 2× SDS Laemmli sample buffer at 95 °C for 5 min. After co-IP, samples were analyzed via immunoblotting with anti-CBP (1:500; 7389S, Cell signaling Technology), anti-β-catenin, and anti-Smad3 antibodies. Anti-mouse IgG VeriBlot for IP secondary antibody (Abcam, Cambridge, UK) was used for β-catenin detection. A secondary antibody in the ImmunoCruz IP/WB Optima F System (Santa Cruz Biotechnology) was used to detect Smad3.

### 4.9. Measurement of Hydroxyproline Contents in the Liver

The cryopreserved hepatic tissue (100 mg) was pulverized using a multibead shocker (MB2200; Yasui kikai, Osaka, Japan). After adding 1 mL of deionized water, the sample was subjected to three freeze–thaw cycles. The resulting homogenates were sonicated for 15 min with a 15 s on/off cycle using a Bioruptor (Cosmo Bio Co., Ltd., Tokyo, Japan) at a power output of 250 W. A small aliquot of the homogenate was used to quantify the protein concentration. Subsequently, 100 µL of the homogenate was combined with an equivalent volume of 12 N HCl and hydrolyzed at 120 °C for 3 h. After centrifugation at 12,000× *g* for 5 min, 10 μL of the supernatant was evaporated to remove HCl. Hydroxyproline content was measured following treatment with a standard 1M Ehrich’s solution at 65 °C for 20 min, followed by oxidation in a 50 mM Chloramine T (NACALAI TESQUE, INC.) solution at room temperature (18–25 °C) for 25 min. Absorbance was measured at 560 nm using a Sunrise Rainbow plate reader (Tecan Group Ltd., Zurich, Switzerland).

### 4.10. Histological Analyses

The 3-μm-thick sections were prepared from paraffin embedded liver tissues and stained with the picrosirius red staining kit (Polysciences, Warrington, PA, USA) according to the manufacturer’s instructions. The 2-μm-thick sections were stained with Azan via a conventional methodology. Ten distinct fields from each section were randomly captured at 100-fold magnification, followed by quantification of the stained area relative to the total area using a BZ-X Analyzer (Keyence Corporation, Osaka, Japan).

### 4.11. Collagenase Activity Assay

Collagenase activity was assessed as previously described with minor modifications [56]. Liver tissue was homogenized in a buffer consisting of 50 mM Tris-HCl (pH 7.4), 0.2 M NaCl, 5 mM CaCl_2_, and 0.1% Triton X-100. The supernatant was collected via centrifugation. A 40-µg sample was treated with 100 µg/mL of trypsin (FUJIFILM Wako Pure Chemical) at 28 °C for 1 h, after which the reaction was inhibited with 500 µg/mL of trypsin inhibitor (FUJIFILM Wako Pure Chemical). The type I collagenase activity of the resultant sample was measured using a Type I collagenase assay kit (Cosmo Bio) according to the manufacturer’s instructions. Collagenase activity was evaluated using an alternative commercial assay kit supplied by Abcam (Collagen Degradation/Zymography Assay Kit (fluorometric)) according to the manufacturer’s instructions.

### 4.12. Statistical Analyses

Data distribution was evaluated using the Shapiro–Wilk normality test. For statistical analysis of parametric data, one-way ANOVA, followed by Dunnett’s or Tukey multiple comparison post-hoc tests, was employed for comparisons among more than two groups; the two-tailed unpaired Student’s *t*-test was used for comparisons between two groups unless specified otherwise. For non-parametric data, the Steel test was applied for comparisons involving more than two groups, and the Mann–Whitney U test was employed for two-group comparisons. Statistical significance was set at *p* < 0.05. SPSS version 27 (IBM Corp., Armonk, NY, USA) and MEPHAS (https://alain003.phs.osaka-u.ac.jp/mephas/, accessed on 21 June 2023 and 3 July 2023) were used for statistical analyses.

## Data Availability

The original contributions presented in this study are included in the article/Supplementary Materials. Further inquiries can be directed to the corresponding author.

## Supplementary Materials

The following supporting information can be downloaded at https://www.mdpi.com/article/10.3390/ijms27219522/s1.

## Abbreviations

ECM, extracellular matrix; HSCs, hepatic stellate cells; MMP, matrix metalloproteinase; CCl_4_, carbon tetrachloride; HFD, high-fat diet; CBP, CREB-binding protein; MASH, metabolic dysfunction-associated steatohepatitis; DAMPs, danger-associated molecular patterns; TGF-β, transforming growth factor-β; CBP, CREB-binding protein; α-SMA, alpha-smooth muscle actin; COL1A1, collagen type 1α1; CD, control diet; TCF-4, T-cell factor-4; LEF, lymphoid enhancer-binding factor; qPCR, quantitative PCR; CTGF, connective tissue growth factor; UDCA, ursodeoxycholic acid; LOX, lysyl oxidase; LOXL1, lysyl oxidase like-1; TIMP1, tissue inhibitor of metalloproteinase-1; P4HA, prolyl 4-hydroxylase subunit alpha; HCC, hepatocellular carcinoma.
