# Supplementary material for: Therapeutic Targeting of Hepatic Stellate Cell Inactivation and Collagen Degradation by IC-2 Mitigates Liver Fibrosis
Source: Int J Mol Sci. 2026 Oct 24;27(21):9522. doi: 10.3390/ijms27219522 (PMC13763049; doi:10.3390/ijms27219522)
Supplement: Supplementary file 1 [file ijms-27-09522-s001.zip › ijms-4624434-supplementary.pdf]

Supplemental Information

Therapeutic targeting of hepatic stellate cell inactivation and collagen degradation by IC-2 mitigates liver fibrosis

Noriko Itaba[1,2*], Ken-ichiro Abe[1,†], Yohei Kono[1,‡], Hiroki Shimizu[1,§], Hiroyuki Oka[3,¶], Minoru Morimoto[3,||], Daisuke Nanba[1], Goshi Shiota[1,2]

Supplemental Figures

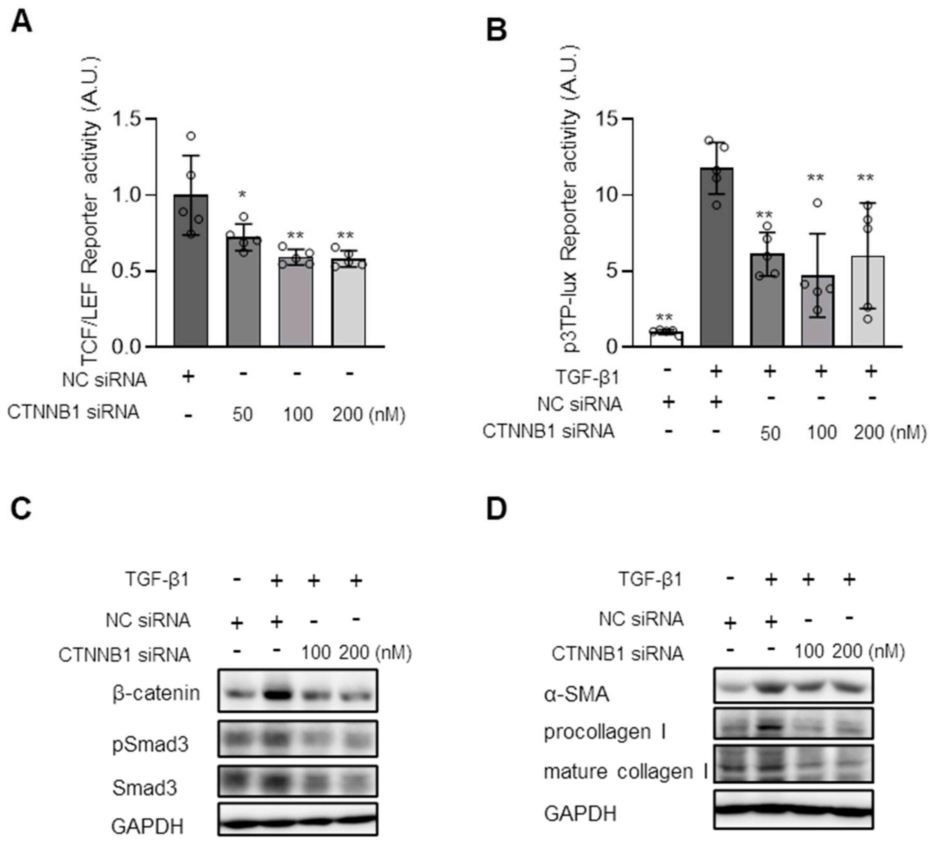

Supplemental Figure S1. Effects of siRNA-mediated β-catenin knockdown in human LX-2 hepatic stellate cells (HSCs).

(**A**) Wnt/β-catenin signal activity after β-catenin siRNA transfection, as measured using the TCF/LEF reporter lentivirus. (**B**) TGF-β/Smad signal activity following β-catenin siRNA treatment, as assessed with the p3TP-lux reporter plasmid. (**C**) Western blots of β-catenin, Smad3, and phosphorylated Smad3 after β-catenin siRNA intervention. (**D**) Western blots of α-SMA, procollagen-I, and mature collagen-I. Statistical analysis was performed using Tukey's test (* $p < 0.05$, ** $p < 0.01$, relative to negative control siRNA, n = 5).

A

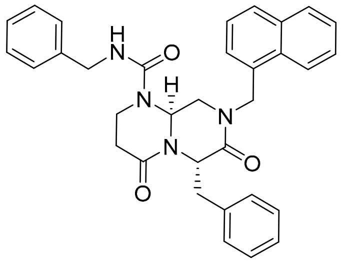

IC-2

B

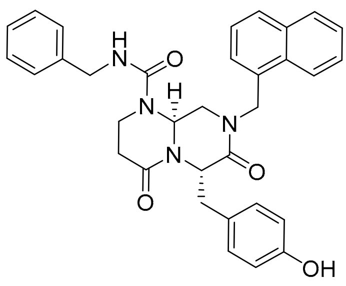

ICG-001

Supplemental Figure S2. Chemical structures of IC-2 and ICG-001.

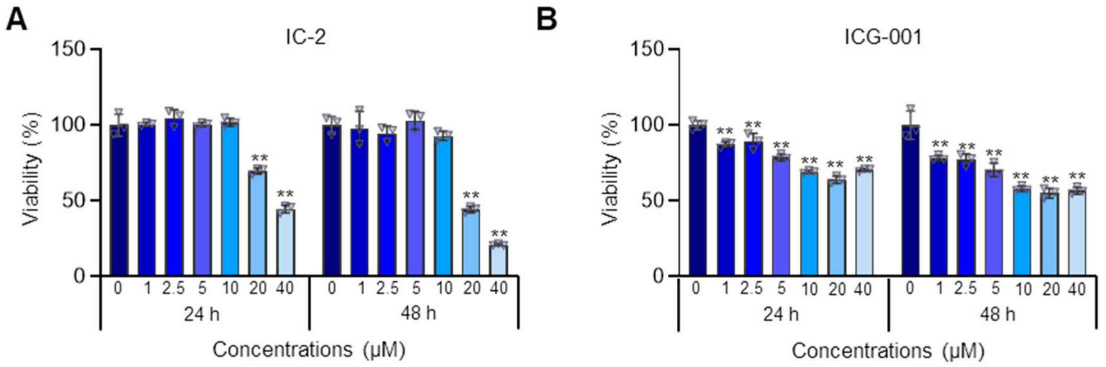

Supplemental Figure S3. Effects of IC-2 and ICG-001 on the viability of human LX-2 cells. The WST assay was performed 24 and 48 h after treatment with IC-2 (**A**) or ICG-001 (**B**). Statistical analysis was conducted using Dunnett's test (** $p < 0.01$ compared with 0 μM, n = 3).

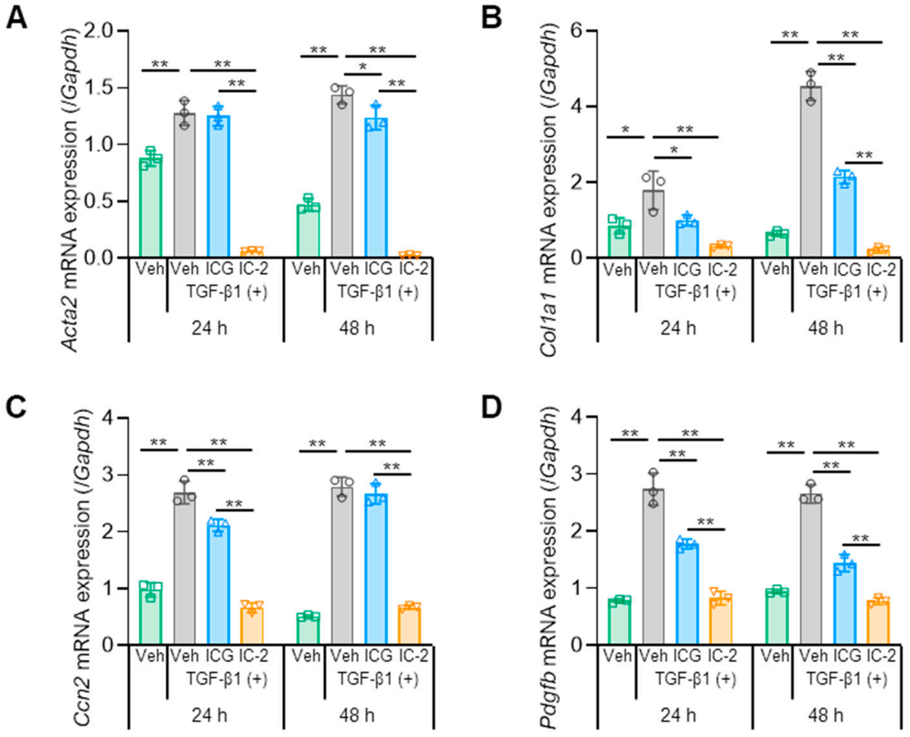

Supplemental Figure S4. qPCR analysis for markers of putative HSC activation.

qPCR was performed 24 and 48 h after treatment with IC-2 or ICG-001 in the presence of TGF-β. Relative expression levels of (**A**) *Acta2*, (**B**) *Col1a1*, (**C**) *Ccn2* (CTGF), and (**D**) *Pdgfb* were normalized to those of *Gapdh*. Statistical analyses were performed using Tukey's test (* $p < 0.05$, ** $p < 0.01$, n = 3).

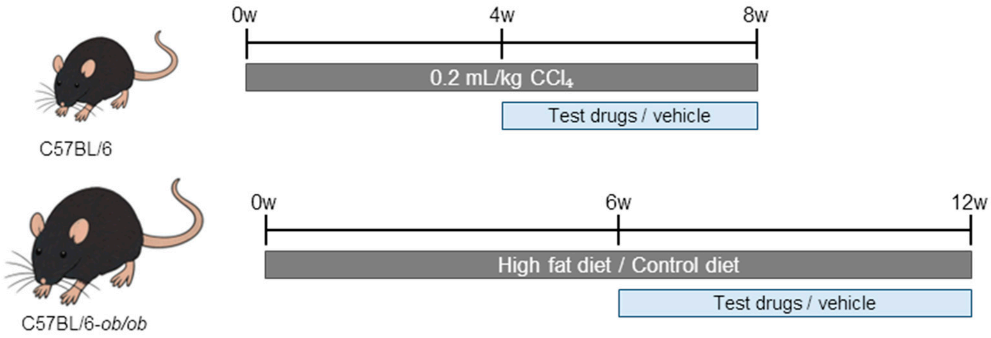

Supplemental Figure S5. Design of the animal experiments.

CCl_4-induced liver fibrosis model: C57BL/6 mice were intraperitoneally administered CCl_4 three times per week for four weeks, followed by intraperitoneal administration of either the drug or vehicle three times per week for an additional four weeks; CCl_4 was administered on the following day. High-fat diet-fed liver fibrosis model: C57BL/6-*ob*/*ob* mice were fed a high-fat diet for six weeks, followed by intraperitoneal administration of either the drug or vehicle three times per week for an additional six weeks.

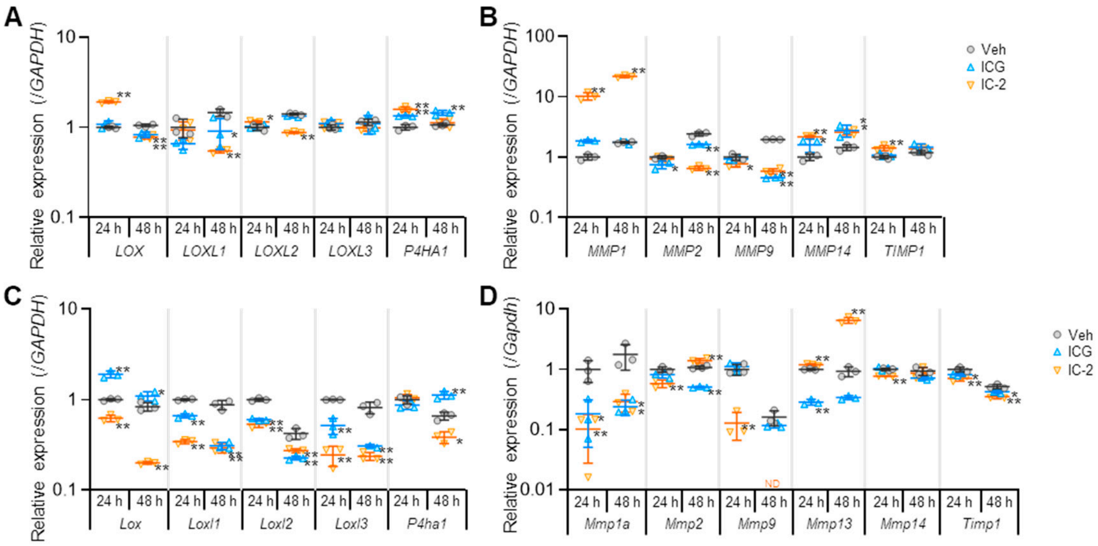

Supplemental Figure S6. qPCR analysis of fibrogenesis- and fibrolysis-related genes in human and mouse HSCs in the absence of TGF-β treatment.

qPCR was performed at 24 and 48 h after treatment with IC-2 or ICG-001. (**A** and **B**) Expression of (**A**) fibrogenesis- and (**B**) fibrolysis-related genes in the human HSC line LX-2. (**C** and **D**) Expression of (**C**) fibrogenesis- and (**D**) fibrolysis-related genes in primary mouse HSCs. *GAPDH* and *Gapdh* served as internal controls. Statistical analyses were performed using Dunnett's test (* $p < 0.05$, ** $p < 0.01$, relative to DMSO treatment; n = 3).

Supplemental Table S1. Body weight, liver weight, and biochemical data in the serum of CCl_4-induced liver fibrosis mice

Group		Body weight (g)	Liver weight (g)	Liver/body weight ratio	ALT (IU/L)	AST (IU/L)	T-bil (mg/dL)
vehicle		25.2 ± 1.0	1.36 ± 0.10	0.054 ± 0.004	80.3 ± 31.6	96.3 ± 45.5	0.14 ± 0.07
Glycyrrizin	165 mg/kg	24.8 ± 0.8	1.35 ± 0.08	0.055 ± 0.003	114.9 ± 66.7	123.0 ± 63.8	0.21 ± 0.08
ICG-001	5 mg/kg	24.8 ± 1.3	1.29 ± 0.11	0.052 ± 0.004	72.4 ± 34.8	108.1 ± 52.9	0.33 ± 0.12 **
IC-2	5.3 mg/kg	25.0 ± 1.1	1.26 ± 0.09	0.050 ± 0.002 *	74.1 ± 17.4	102.8 ± 36.0	0.47 ± 0.19 **
	1.6 mg/kg	25.2 ± 1.2	1.29 ± 0.08	0.051 ± 0.002	72.6 ± 14.2	119.8 ± 42.5	0.31 ± 0.13 **
	21.2 mg/kg	24.9 ± 1.5	1.25 ± 0.14	0.050 ± 0.004 *	78.3 ± 31.6	116.2 ± 53.4	0.28 ± 0.12 *
CCl_4(-)		27.5 ± 1.7 **	1.35 ± 0.07	0.049 ± 0.002 *	5.9 ± 1.4 **	15.9 ± 3.3 **	0.08 ± 0.02

All data are expressed as the mean ± SD of 11 to 12 mice for each group (except for n = 6 for the CCl_4(-) group).
*$p < 0.05$, **$p < 0.01$, compared with the vehicle (One-way ANOVA followed by Dunnett's post hoc test).

Supplemental Table S2. Body weight, liver weight, and biochemical data in the serum of MASH model mice

Group		Body weight (g)	Liver weight (g)	Liver/body weight ratio	ALT (IU/L)	AST (IU/L)	T-bil (mg/dL)	Blood glucose (mg/dL)
vehicle		59.7 ± 2.0	4.76 ± 0.32	0.080 ± 0.005	192.8 ± 55.9	217.1 ± 41.5	0.29 ± 0.17	349.8 ± 111.3
UDCA	150 mg/kg	56.8 ± 2.7	3.50 ± 0.50 **	0.061 ± 0.006 **	95.0 ± 20.4 **	159.0 ± 33.9	0.53 ± 0.31	245.9 ± 60.6 **
ICG-001	5 mg/kg	60.5 ± 3.9	4.61 ± 0.97	0.076 ± 0.013	154.8 ± 68.9	171.5 ± 60.0	0.19 ± 0.06	353.3 ± 119.0
IC-2	5.3 mg/kg	60.3 ± 3.4	4.65 ± 0.43	0.077 ± 0.006	145.0 ± 31.7	170.5 ± 23.7	0.24 ± 0.28	410.0 ± 60.0
	1.6 mg/kg	60.8 ± 3.0	5.26 ± 0.67	0.086 ± 0.008	157.3 ± 46.5	193.8 ± 69.0	0.25 ± 0.24	403.4 ± 66.8
	21.2 mg/kg	60.7 ± 4.9	5.15 ± 0.51	0.085 ± 0.006	192.2 ± 75.6	198.0 ± 63.1	0.38 ± 0.40	401.9 ± 91.1
CD		48.3 ± 2.7 **	3.65 ± 0.75 **	0.076 ± 0.016	105.9 ± 40.0 **	190.1 ± 67.4	0.25 ± 0.12	212.0 ± 69.6 **

All data are expressed as the mean ± SD of 10 to 11 mice for each group.

*$p < 0.05$, **$p < 0.01$, compared with the vehicle (One-way ANOVA followed by Dunnett's post hoc test).

Supplemental Table S3. Primer sequences for qRT-PCR analyses.

Genes	Sense (5' – 3')	Antisense (5' – 3')	Species
ACTA2	CTGTTCCAGCCATCCTTCAT	CCGTGATCTCCTTCTGCATT	Human
COL1A1	CCTCCAGGGCTCCAACGAG	TCAATCACTGTCTTGCCCCA	Human
LOX	GCCTCAGGCTGCACAATTTC	TCAGAACACCAGGCACTGATTT	Human
LOXL1	GTCGCTACGTTTCTGCAACA	GCTTTGGAAGGGGAGAGATT	Human
LOXL2	GCCACATAGGTGGTTCCTTCA	GAAGATGTGGTGTGGCCTGA	Human
LOXL3	CGGATGTGAAGCCAGGAAACT	GGCATCACCAATGTGGCA	Human
P4HA1	GCTCTCTGGCTATGAAAATCCTG	GTGCAAAGTCAAAATGGGGTTC	Human
MMP1	TCAACCAGGCCCAGGTATT	AGCTGCAACACGATGTAAGT	Human
MMP2	ACCTACACCAAGAACTTCCG	GGTCAATGTCAGGAGAGGC	Human
MMP9	TCCCTGGAGACCTGAGAAC	CCACCCGAGTGTAACCATAG	Human
MMP14	CCTACCGACAAGATTGATGCTG	AAACGGTAGTACTTGTTTCCACG	Human
TIMP1	CTGTTGGCTGTGAGGAATGC	GAGCTGGTCCGTCCACAA	Human
GAPDH	AGCCACATCGCTCAGACAC	GCCCAATACGACCAAATCC	Human
Acta2	GTTCAGTGGTGCCTCTGTCA	ACTGGGACGACATGGAAAAG	Mouse
Col1a1	AAACCCTCCCCTCTTGCTGC	TTGTTTCCTCCCAGCCCAGG	Mouse
Ctgf	TGACCTGGAGGAAAACATTAAGA	AGCCCTGTATGTCTTCACACTG	Mouse
Pdgfb	CCTGAGGAACTGTATGAAATGCT	CAGCTCAGCCCCATCTTC	Mouse
Pparg	GCTGAACGTGAAGCCCATCG	TTCTGGAGCACCTTGGCGAA	Mouse
Lox	TCCGACGACAACCCCTATTA	AGGTCCGGGAGACCGTACT	Mouse
Loxl1	GAGTGCTATTGCGCTTCCC	GGTTGCCGAAGTCACAGGT	Mouse
Loxl2	TTCTGCCTGGAGGACACTGAGT	TCGGTGATGTCTATCCACTGGC	Mouse
Loxl3	AGGGTGGACCCATAGTGCC	GCATTCTGTCACACTTCCTTCA	Mouse
P4ha	CCCAGTCAGGTCTGCTATTC	GGAACAGTCTCTGGACAACC	Mouse
Mmp1a	TGCCATCGCGAGAGCCTTTA	TCACCATGGTCTCCTCTGTGG	Mouse
Mmp2	TTCCCCCGCAAGCCCAAGTG	GAGAAAAGCGCAGCGGAGTGACG	Mouse
Mmp8	ACTCCACTGATCCTGGTGCC	TGGGCCCAGTAGGTTGGATG	Mouse
Mmp9	AGTGGGACCATCATAACATCACAT	TCTCGCGGCAAGTCTTCAG	Mouse
Mmp13	GCTGGTCAGTCGCCCTTTT	TAAGGAAAGCAGAGAGGGATTAACA	Mouse
Mmp14	CGTTCGCTGCTGGACAAGG	GACTGAGAAGGGAGGCTGGAG	Mouse
Timp1	AGGTGGTCTCGTTGATTTCT	GTAAGGCCTGTAGCTGTGCC	Mouse
Timp2	ACGCTTAGCATCACCCAGAAG	GGACAGCGAGTGATCTTGCA	Mouse
Timp3	GCGGAAGCGTGCACATG	GCTTCTTTCCCACCACTTTGG	Mouse
Gapdh	TGAGTATGTCGTGGAGTCTAC	TGGACTGTGGTCATGAGCC	Mouse

Supplemental Table S4. Shared and unique promoter motifs identified in human and murine collagenase genes by JASPAR analysis (within 3 kb promoter regions).

Matrix ID	Motif Name	Class	Family	human MMP1	murine Mmp13	human MMP13	murine Mmp1a
UN1167.1	ATF3::GABPA	Basic leucine zipper factors (bZIP)::Tryptophan cluster factors	Fos-related::Ets-related	+	+	-	-
UN1152.1	TFAP4::GABPA	Basic helix-loop-helix factors (bHLH)::Tryptophan cluster factors	bHLH-ZIP::Ets-related	+	+	-	-
MA0450.1	hkb	C2H2 zinc finger factors	Three-zinc finger Kruppel-related	+	+	-	-
MA1461.2	sv	Paired box factors	Paired domain only	+	+	-	-

Motif searches were conducted using JASPAR 2026 across the 3,000 bp upstream promoter regions of human *MMP1* (ENST00000315274.7), human *MMP13* (ENST00000260302.8), murine *Mmp13* (ENSMUST00000015394.10), and murine *Mmp1a* (ENSMUST00000217651.3) with a relative score threshold ≥ 0.80. (+) indicates presence, and (-) indicates absence.

Methods

WST assay

Cell viability was assessed after treatment with IC-2 or ICG-001 using Cell Counting Kit-8 (DOJINDO LABORATORIES, Kumamoto, Japan) according to the manufacturer's instructions.

Biochemical tests

Serum samples were collected from the inferior vena cava and stored overnight on ice. The serum was isolated by centrifugation at 2,000 ×*g* for 20 min. Serum aminotransferase levels were measured using the Transaminase CII test (FUJIFILM Wako Pure Chemical Industries Ltd.) according to the manufacturer's instructions. Total bilirubin levels were quantified using a QuantiChrom Bilirubin Assay Kit (BioAssay Systems LLC, Hayward, CA, USA).

In Silico Promoter Analysis

In silico transcription factor binding motif analysis was performed using the JASPAR 2026 database (https://jaspar.elixir.no/) [1]. Promoter sequences spanning 3,000 bp upstream of the transcription start sites (TSS) for human MMP1 (ENST00000315274.7), human MMP13 (ENST00000260302.8), murine Mmp13 (NM_008607.2), and murine Mmp1a (NM_032006.3) were retrieved. Motif matching was executed across both strands using position weight matrices (PWMs) in JASPAR CORE (vertebrates) and UNVALIDATED collections. A relative score threshold of ≥ 0.80 was applied to identify predicted transcription factor binding sites and composite motifs uniquely shared or differentiated among species.

References

1. Ovek Baydar, D.; Rauluseviciute, I.; Aronsen, D.R.; Blanc-Mathieu, R.; Bonthuis, I.; de Beukelaer, H.; Ferenc, K.; Jegou, A.; Kumar, V.; Lemma, R.B.; Lucas, J.; Pochon, M.; Yun, C.M.; Ramalingam, V.; Deshpande, S.S.; Patel, A.; Marinov, G.K.; Wang. A.T.; Aguirre. A.; Castro-Mondragon, J.A.; Baranasic, D.; Chèneby, J.; Gundersen, S.; Johansen, M.; Khan, A.; Kuijjer, M.L.; Hovig, E.; Lenhard, B.; Sandelin, A.; Vandepoele, K.; Wasserman, W.W.; Parcy, F.; Kundaje, A.; Mathelier, A. JASPAR 2026: expansion of transcription factor binding profiles and integration of deep learning models. Nucleic Acids Research 2026 54 (D1), D184-D193. DOI: 10.1093/nar/gkaf1209.
